# Slowing dementia symptoms – a qualitative study on attitudes and experiences of general practitioners in Germany

**DOI:** 10.1080/13814788.2022.2037550

**Published:** 2022-03-07

**Authors:** Julian Wangler, Michael Jansky

**Affiliations:** Centre for General and Geriatric Medicine, University Medical Centre Mainz, Mainz, Germany

**Keywords:** Dementia care, general practitioner, family doctor, dementia diagnostics, intervention, treatment, management

## Abstract

**Background:**

There is currently no cure for dementia but general practitioners (GPs) have therapeutic options available to counteract the progression of mild cognitive impairment, including drug and non-drug treatment. So far, few studies have investigated treatment strategies preferred by GPs.

**Objectives:**

This study aimed to gain an overview of GPs’ attitudes towards influencing the progression of dementia, their involvement regarding dementia prevention and perceived effective approaches. It also elucidated the challenges experienced by GPs and desired optimisation measures towards reinforcing secondary prevention.

**Methods:**

Between June 2020 and March 2021, 64 semi-standardised interviews amongst GPs were conducted in all federal states of Germany. Thirty interviews were carried out in person and 34 by phone. The data were analysed according to qualitative content analysis.

**Results:**

Many interviewees see great importance in secondary dementia prevention and believe they could make an effective contribution, some of them using non-drug approaches. GPs play a role in guiding patients and relatives towards support services. Some doctors consider drug treatment as the only option towards influencing the progression of dementia, showing low expectations on self-efficacy. Interdisciplinary collaboration is a frequent challenge, which often conflicts with a coherent treatment strategy.

**Conclusion:**

Many GPs feel confident about influencing the progression of dementia and believe they can intervene effectively, using various (non-drug) treatment measures and referrals to support services. GPs perceive challenges, including obstacles in interdisciplinary collaboration and negative impacts after drug administration. To improve the conditions for GP intervention, it depends on expanding interdisciplinary collaboration and care strategies.

KEY MESSAGES

- GPs can play a crucial role in identifying and managing dementia.
- Many GPs see themselves in a position to influence the progression of dementia positively.
- Introducing patients and caregivers to support services can stabilise the home care setting.
- Interdisciplinary care strategies and structures seem important in effectively managing dementia.

## Introduction

Dementia is the leading cause of dependency and disability among older people in Europe and currently affects around ten million people in this region; its prevalence is expected to double by 2030 [1]. Around 1.7 million people have contracted dementia in Germany in 2019 [1].

Evidence-based guidelines assume that diagnosing dementia as early as possible plays a crucial part in setting a timely course for effective care. Even if there is no cure for dementia yet, there are indications that targeted intervention in the early stages can impact the course of the disease [2–8]. Apart from treatment, it is important to prepare patients and caregivers in good time and provide support [6,8–10]. The DSM V categorisations of dementia as neurodegenerative disorders emphasise that behavioural and psychological symptoms determine treatment options and patterns of care [11]. Subsequently, general practitioners (GPs) play a major part in recognising cognitive changes early on and managing dementia patients [3,5–8,12,13].

There are various options conceivable in primary care towards counteracting the progression of mild cognitive impairment [8,9,12]. Individual studies have shown positive effects for specific measures. Various authors have emphasised the importance of multimodal treatment rooted in primary care, combining drug and non-drug treatment strategies at different levels [4,5,13]. Adjuvant drug treatment mainly comprises anti-dementia drugs, sleeping pills and sedatives [14]. Non-drug approaches include physical activation, physiotherapy and cognitive training to help maintain everyday skills [7]. A study in Germany showed that occupational therapy improves the liveability of dementia patients and reduces mental deterioration [2]. By guiding patients and caregivers towards counselling and support services, GPs may contribute to maintaining good quality of life [8–10]. Guidelines advocate specific focus on needs-based management, support and psychosocial stabilisation beyond drugs to delay the disease’s progression [3,4,7].

Beyond the work of the individual GP, there is a need for cross-sectoral structures in outpatient dementia care in many European countries [5,13–16]. The lack of effective structures for outpatient crisis intervention usually leads to rapid hospital admission in crises [17]. Model projects striveng to strengthen the integration of GP-based dementia care in interdisciplinary and regional support networks [18].

An occasional criticism regarding management in primary care is that treatment in practice lags behind the guidelines [4,5]. Studies have shown that this is connected to primary care not identifying early dementia symptoms [19,20]. Another finding has been that GPs are not always sufficiently aware of diagnostic and care options [3,5,13,14,21,22]. Reasons for this reluctance include scarcity of time and resources alongside low expectations on effectiveness due to this subjective lack of treatment options [21,23,24]. Beyond these findings, only a few studies are taking an exploratory approach towards investigating those treatment measures that GPs prefer in managing dementia and their experience with these measures [22].

### Research aims

The present study dealt with the question of what attitudes GPs have with regard to the possibility of influencing the course of dementia. We determined how involved GPs are in the therapeutic management of dementia and what intervention approaches they perceive as effective in counteracting the progression of dementia. We also obtained information about the challenges GPs experienced in managing dementia patients and about improvements GPs consider useful for effective therapeutic management of dementia in primary care.

## Methods

### Concept of the study

Since little is known about attitudes and behavioural patterns amongst GPs with regard to the therapeutic management of dementia, there is a need for a broader exploration of this issue. Consequently, a qualitative approach with semi-structured interviews appeared most appropriate. This study focussed on the early to intermediate stage of dementia.

### Recruitment and sampling

First, a pool of 448 potential contact addresses was set up, using doctor finder search engines made available by the Associations of Statutory Health Insurance Physicians (Kassenärztliche Vereinigungen) of the individual federal states. The pool included a wide range of GPs’ offices across all of Germany’s 16 federal states (28 per state). We then began recruiting the sample.

Eighty-four physicians from the pool of contact addresses were contacted *via* telephone or e-mail. These doctors were selected based on various criteria that were intended to ensure that a broad spectrum of GPs is represented in the sample (among other things, form of practice, practice type, further education background). The aim was to have each federal state equally represented in the study regardless of the number of inhabitants, improving coverage especially in rural and sparsely populated regions. In selecting the target number of four GPs per state, we ensured that gender and environment (rural/urban) were equally represented. Besides, we focussed on achieving a broad geographical distribution of GPs’ offices in the individual federal states while taking different age groups as well as various qualifications into account (Table 1).

**Table 1.** Sociodemographic characteristics of the sample (*N* = 64).

Office type	
Joint practice	24 (38%)
Single office	40 (63%)
Office environment	
Small towns or rural communities	20 (31%)
Medium-sized towns	24 (38%)
Cities	20 (31%)
Employment type	
Practice owner	48 (75%)
Employed	16 (25%)
Age	
Mean (range)	54 years (23 years)
Gender	
Female	32 (50%)
Previous knowledge and qualifications in the field of dementia	
Training in dementia diagnostics and care	11 (17%)
Regular participation in quality circles	9 (14%)
Further geriatric training	7 (11%)

Sixty-four interviews were finally carried out. The interviews took place between June 2020 and March 2021 and were conducted by two general practice researchers, each conducting half of the interviews. Thirty interviews were conducted in person and 34 by phone (40–70 min). Table 1 provides an overview of the participating samples.

### Investigation tools

The interview guides were developed based on a review of the literature (see background; mainly, the contribution by Radisch et al. [4] played a prominent part as it presented a strategy for integrated, GP-based dementia care). In addition, we used references taken from several preliminary studies carried out by the authors in developing the guide; these studies focussed on GPs’ attitudes, behavioural patterns and competence indicators in identifying dementia [20,22]. In the course of the first interviews, the instrument was further specified.

The interview guide consisted of 22 questions with several sub-questions and primarily focussed on the following topics: Perception of roles in dementia care; perceived options towards influencing dementia in primary care; intervention approaches used and resulting experience; challenges found in treatment management; importance of and collaboration with regional assistance and support networks; approaches towards optimising dementia prevention for GPs; assessment of personal competence and previous experience in the field (Appendix 1).

### Data analysis

After data collection, the team evaluated the resulting transcripts using qualitative content analysis according to Mayring [22]. This first entailed pinpointing the key statements, followed by further abstraction and summarisation, finally leading to a categorised system closely based on the interview guides and repeatedly reviewed and modified as necessary during evaluation. Our focus lay on forming logical categories from the various opinions and experiences.

Theoretical saturation became apparent after 53 interviews. However, we had set the condition that the same number of interviews should be conducted in each state, so we had 64 interviews (four per state).

### Ethics

No sensitive patient data were gathered during this study or clinical tests performed. All 64 expert interviews with GPs were strictly anonymised. The Ethics Commission of the State of Rhineland-Palatinate, Germany, informed us that approval by an ethics committee was not necessary. The researchers identified the participants and requested their written consent to participate in the study.

## Results

### Attitude and involvement regarding dementia prevention

Of the 64 interviewees, 61 stated that they provided dementia diagnostics in their own offices, 33 indicated that they were occasionally or more often involved in the treatment of dementia patients, and 15 emphasised that they more closely followed on from the approach taken by the specialist involved in the case. The interviewees showed a noticeably wide range of opinions on the benefit of specific treatment for dementia.

*Of course GPs need to do all they can to positively influence on disease progression. I’m sure it can be done. (I-18m)*

*There are many options to try out and we should go through all the possibilitiesavailable, but dementia is difficult to predict. (I-1f)*

*I rather doubt that any treatment can make a real difference to this medical condition. (I-36m)*

About half of the interviewees felt confident about the medical options available to counter the progression of dementia. They stated that it was indeed possible to delay dementia effectively because ‘solutions were developed specifically for that particular patient to increase patient well-being’ (I-39m). In contrast, the other half of the interviewees (31) showed scepticism on the prospect of effective dementia prevention. Conspicuously, this was frequently associated with negative perceptions of effectiveness and self-efficacy; one GP emphasised that aiming to treat dementia was practising ‘nothing more than deficiency management’ (I-33f). Other interviewees focussed on their own feelings of powerlessness and negative experience in the past:

*Of course you want to help. However, we as doctors reach our limits all too often with this clinical condition. Getting consumed in the idea of achieving something at a fundamental level will only lead to great disappointment. (I-2m)*

### Dementia prevention measures and the effects seen

Most doctors emphasised that keeping patients in their home environment should be an aim, staving off admission to a hospital or nursing home as far as possible. Many doctors have reported ‘major long-term decompensation in dementia sufferers’ (I-13f) after being removed from their familiar surroundings.

Two groups stood out among those reporting that they frequently or occasionally participated in dementia treatment for their patients. The first group of doctors only saw prevention in terms of drug treatment.

*I can’t think of any real options beyond prescribing existing medicines such as anti-dementia drugs, psychotropic drugs etc. (I-22m)*

The second group went well beyond this by combining drug treatment with other measures, in some cases preferring other forms of treatment over dementia-specific drug treatment.

The interviews revealed a wide range of supporting measures (Table 2). These GPs were convinced of intrinsic motivation and creativity, targeted perception, attention and memory training and also relaxation techniques and encouraging exercise, amongst other things as influencing factors in at least slowing down the progression of dementia. They held that dementia ‘can only be halted effectively in a dynamic context, that is, including physical, cognitive and psychosocial methods’ (I-14f). They also placed great value on involving relatives in intervention.

**Table 2.** Frequently reported intervention approaches in dementia prevention.

Preferred measure	Number of interviewees	Exemplary sample quote
Methodical treatment for co-morbidities	29	‘*You have to practice risk management and should treat things like delirium, heart failure and diabetes thoroughly as this clearly reduces dementia symptoms.*’ (I-41m)
Physical activation: Physical exercise and sport, physiotherapy and occupational therapy	17	‘*The ability to maintain everyday bodily functions goes hand in hand with cognitive function. […] People simply stay fresh and lively for longer.*’ (I-15w)
Cognitive training and stimulation	15	*‘Methodically training awareness, attention and memory is highly beneficial.’* (I-27m)
Creative and artistic therapy	9	‘*If you ask me, the best approach is to activate people’s creativity. Play music, do something artistic that keeps you mentally occupied, or go dancing.*’ (I-60w)
Psychosocial support, psychoeducational intervention, psychiatric treatment	8	‘*You can train patients how to learn to deal with the disease in a positive way. Inner balance and deep relaxation play a major role.*’ (I-35w)
Change in diet	7	‘*Diet is given too little attention in its effects on dementia. Diet is a lifestyle matter with a direct impact on your health, especially if you’re a high-risk patient.*’ (I-15w)
Intervention involving relatives: Behaviour management, coping therapy, options for relief	20	‘*Relatives are often the real influencing factor. You have to do everything you can to keep these people stable and bolster their resilience. Patients also depend on it.*’ (I-25w)

With this in mind, the corresponding physicians saw themselves as a conduit to various assistance and support services, some also using local networks. Intensive collaborations with care support centres, care services, therapists (especially physiotherapists), health centres and self-help groups were frequent.

The interviewees reported how the ‘resignation and potentially depressive impact of a dementia diagnosis’ could be ‘more than compensated for in the long term’ using a well-designed combination of non-drug treatments (I-46m). Patients and relatives needed to ‘find a new balance for themselves under these new conditions’ (I-25f). GPs were in a position to provide support as needed in ‘paving the way for access to support structures’ (I-42m) to ‘reduce the strain while maintaining quality of life and emphasising the opportunities in subjective perception’ (I-62f). ‘Confidence in daily life’ would lead to ‘more mental balance and stability’ amongst dementia patients and their relatives (I-35f). This was ‘one of the greatest indicators of a more favourable course of dementia’ according to their own assessment (I-27m).

Many of the interviewees emphasised the difficulty of making an objective assessment of the effectiveness of dementia treatment. Many were convinced that this effectiveness ‘always depends on the individual […] so more like real secondary prevention’ (I-39m).

### Challenges experienced

Some GPs addressed issues of not having a good overview of health care, support services to provide assistance and counselling for dementia patients and their relatives regarding the perceived hurdles and challenges in dementia prevention. These issues were especially prevalent amongst the group that did not consider the use of non-drug treatments.

Medication and interdisciplinary collaboration were also frequently cited, often directly. Some physicians expressed scepticism on the benefits of established dementia drugs ‘in a very general sense’ (I-36m) from their own experience.

*I’ve seen many patients over the years taking these powerful psychiatric and anti-dementia drugs. These drugs haven’t really seemed compelling or promising. (I-53f)*

Especially those doctors mainly focussed on drug treatment showed noticeable dissatisfaction with treatment efficacy.

*You can certainly use something for treatment, but any expectations or hopes wouldbe misplaced as they won’t live up to them anyway. (I-45m)*

Some interviewees reported side effects such as personality changes (mood swings, disruption of the day–night rhythm) after administering neuroleptics, and one ‘became very cautious’ about prescribing drugs (I-60f).

Most of the GPs involved in treatment were critical of interdisciplinary collaboration. Apart from lack of availability of psychiatric or neurological specialists and long waiting times, the results show a tendency amongst specialists to restrict GPs’ scope of activity by prescribing nonsensical prescriptions without consultation or feedback to the GP.

*I often wonder ‘Why that particular drug?’ Psychiatrists pass judgment, and we general practitioners have to run with it, we are railroaded into treatment strategies we wouldn’t have taken. (I-50m)*

Some cases of ‘dire consequences’ from ‘excessive medication and dosages’ (I-42m) led to the above negative effects on psychological integrity. Some of the interviewees that were more focussed on non-drug treatment and targeted psychosocial stabilisation reported conflicted and contradictory treatment strategies by specialists working against their own approaches.

*You sometimes feel that they’re defeating your own efforts at building patients back up. (I-39m)*

However, some interviewees exercised self-criticism in admitting that many GPs did not always consult enough with their specialist colleagues.

*We try to go it alone far too often. This is hardly beneficial in dementia with all the complex fine-tuning the disease requires. (I-1f)*

Another issue facing dementia prevention was its burden on doctor-patient relationships after diagnosis. Dealing with some patients was often challenging due to their reluctance to admit mental deterioration for fear of losing their independence to make decisions; this affected compliance.

### Optimisation approaches

The doctors interviewed raised several points repeatedly when asked about improvements they thought should be made to reinforce the prevention angle of dementia in primary care. The lack of structured cross-disciplinary treatment programmes rooted in primary care received heavy criticism.

*Why aren’t there any dementia disease management programmes? We need programmes to reinforce the interdisciplinary approach. (I-47m)*

Apart from that, several GPs proposed developing an evidence-based, GP-oriented diagnosis and therapy algorithm for dealing with dementia patients. This would help identify symptoms in a targeted way while also creating a better understanding of the next steps in treatment. A widely established and accepted algorithm would be especially effective in encouraging ‘GPs and specialists to pull together’ (I-35f).

Some interviewees exercised self-criticism on the role of primary care in this medical condition, insisting that GPs should invest more time and energy in specialised further training and willingness towards more consistent diagnostics and care for their dementia patients.

## Discussion

### Main findings

The interviews have shown that many GPs see great importance in dementia prevention and believe that they can play an effective part by their own intervention. Many GPs also attach considerable importance to (integrative) therapeutic measures alongside drug treatment. The interviews have, therefore, revealed a wide range of treatment strategies, particularly methodical treatment for co-morbidities, physical exercise and sport as well as physiotherapy and occupational therapy, cognitive training and creative therapy. The GP’s role as a guide through the health care system comes into play in arranging counselling and support services for patients and their relatives, therefore, contributing to their psychosocial stability.

On the other hand, some of the doctors in the sample saw drug treatment as the only option towards influencing the progression of dementia. Low expectations of positive treatment outcomes were prevalent in this group. There was also a tendency to leave treatment planning to specialists and withdraw from active patient management.

The GPs interviewed perceived several challenges when it comes to preventing dementia, including negative impact on personality structure after administering drugs as well as obstacles in interdisciplinary collaboration.

### Comparison with prior work

Overall, the interviews not only confirmed many of the results from preliminary studies [20,22] but also fit in with the general findings for both Germany and other European countries, even if it has to be taken into account that the primary care systems can differ greatly depending on the country. Previous studies have already shown GPs to be in a favourable position to recognise cognitive changes in their patients early on and coordinate treatment; some well-informed and trained doctors and their offices occasionally stand out with their own efforts in optimising early detection of dementia [22]. Even so, there are various signs that the topic is still beset by many obstacles throughout primary care [3,5,9]. Earlier studies have suggested that some GPs see a lack of therapeutic consistency in dementia care due to a focus on purely drug-based treatment; non-drug treatment strategies have only been given limited attention or consideration for clinical application [4,13,20,23,24]. Some studies have also shown that GPs do not always wish to be involved in treating dementia since some of them reveal a deep scepticism about the manageability of dementia as a medical disease [20,22]. In addition, GPs experience uncertainty in the diagnostic and therapeutic process; apart from that, these physicians do not always have the necessary theoretical knowledge or practical skills in this field [3,21–23,25]. Some GPs see relationships with their patients as tense [22,26]. Only one group of GPs guided patients and relatives to support services such as care centres and dementia networks; many are unaware of these resources [10,20].

A systematic review conducted by Low et al. [23] that incorporated studies from different countries has shown massive variation in the willingness of GPs to play an active role in dementia care. Here, decisive factors are beliefs regarding dementia and treatment efficacy, patient circumstances, level of severity and family support as well as the health care system including access to specialists and (diagnostic) services. In addition, GPs do not always consider an approach based on evidence-based guidelines to be feasible or sensible [9].

Some research papers emphasise the importance of non-drug intervention as well as early guidance of patients and their relatives towards support services and the outstanding value of (preventive) stabilisation for family caregivers [4,6,8,10]. This is the only way of achieving mental balance and security for patients in their everyday activities, according to Kurz et al. [27]. Various authors have advocated a multimodal therapeutic approach consisting of occupational and physical therapy as well as patient activation and, where necessary, psychotherapeutic intervention as well as early targeted application of non-drug and drug-based treatment in a holistic approach for patients and their relatives [2,28,29]. This would require transferring expertise in professional and further training programmes.

In line with this, interviewees articulated a desire for outpatient care structures to ensure methodical therapy for dementia patients. A variety of specialist assessments also reflect this [4]. Beyond individual general practice, treatment and interaction structures are lacking that would otherwise enable multi-professional, cross-disciplinary care to provide needs-based and guideline-oriented care for patients with dementia [4,12,16,30]. Studies on case management in dementia care have not yet revealed consistent results but case management seems to be a good factor that might facilitate the needs-based, stepped activation of different health care professionals and thus improve the care of people with dementia [31,32]. The lack of effective outpatient crisis intervention structures often leads to hospital admissions in crises, resulting in serious complications for patients [17]. Patients with dementia should only be admitted to hospital if medically necessary; ideally, treatment should be geared exclusively towards outpatient care as far as possible [7–9]. Targeted training and routine involvement of office staff in primary care play an essential part in early dementia diagnosis and treatment [33,34].

### Strengths and limitations

The study shows that GPs experience a tension between medical and psychosocial needs [13,24].

Even with the heterogeneous sample encompassing all the German federal states, the study has various limitations that need to be addressed. Apart from the limited number of cases, recruitment used regional focus areas. It is also worth considering that more GPs with a specific interest in this topic may have participated due to a bias in the willingness of recruited GPs to take part in the study. Many interviews were conducted by telephone; this may have limited the results’ meaningfulness compared to face-to-face interviews.

This study would be useful in conducting in-depth surveys amongst a larger cohort of GPs, such as a representative survey across Germany; this would allow attitudes and behaviour patterns to be examined more closely with respect to the options for action in primary care. The results may also be included in focus group discussions as necessary to pinpoint issues and behaviour patterns in dementia care with more clarity. In addition, the results from this study could be used in developing best-practice examples and needs-oriented, application-oriented professional and further training programmes.

### Implications

GPs should be encouraged to prioritise primary treatment strategies and coordinate resources in dementia patient care [3,5,8,33]. It would seem important that GPs look beyond drug treatment as the only option for influencing progression of dementia, and also include non-drug approaches to keep quality of life at a high level [7–10,35].

Guiding patients and relatives to regional counselling and support services in good time would make a decisive contribution to successful care and psychosocial stabilisation. Overall, collaboration between GPs and these resources should be reinforced [4,30]. Integrated care strategies and structured treatment programmes seem to play a key role for effective intervention in dementia care but have so far been lacking [13,16,17].

Besides, a more GP-oriented diagnosis and therapy algorithm for dealing with dementia patients would help identify symptoms in a targeted way. This could also help structure collaboration with specialists more effectively.

Targeted professional and further training programmes in Geriatrics can help increase awareness in dementia diagnostics and therapy as well as extend the inventory of knowledge and skills in this field amongst GPs [5,7,25].

## Conclusion

Many GPs see great importance in preventing dementia and perceive themselves in a position to make an effective contribution by their own intervention. The interviews have revealed a wide range of treatment strategies alongside drug treatment, such as treatment for co-morbidities, physical exercise and sport as well as physiotherapy and occupational therapy, cognitive training and creative therapy. Moreover, referring to support services plays an important role. The GPs interviewed perceived challenges, including obstacles in interdisciplinary collaboration and negative impacts after drug administration. To improve the conditions for GP intervention, it depends on expanding interdisciplinary care strategies and cross-disciplinary treatment programmes rooted in primary care.

## Data and materials

Research data are available upon request.

## Supplementary Material

Supplemental material: Interview guideline
