# Supplementary material for: Slowing dementia symptoms – a qualitative study on attitudes and experiences of general practitioners in Germany
Source: Eur J Gen Pract. 2022 Mar 7;28(1):32–9. doi: 10.1080/13814788.2022.2037550 (PMC8903749; doi:10.1080/13814788.2022.2037550)
Supplement: Supplemental material: Interview guideline [file IGEN_A_2037550_SM6807.docx]

**Interview guideline**

- To what extent do you provide **dementia diagnostics** in your office; that is, do you regularly perform dementia tests and diagnoses? Please state any reasons why or why not.
- How involved are you in **therapeutic management** of dementia patients? Please state any reasons why you are or aren’t.
- To what extent do you use the **interdisciplinary dementia guideline (S3)** for orientation in diagnostic and therapeutic decisions? How useful do you find the guideline? (Do you also use other national or international resources or recommendations?)
- How effective do you think targeted treatments are in **delaying or positively influencing** dementia, and to what extent do you think dementia development is irreversible and beyond influence?
- What are your views on **drug treatments** in delaying or positively influencing dementia? What have your experiences and observations been in this respect?
- How useful and effective do you think **non-drug treatments** are in dementia? Which treatments exactly? What have your experiences and observations been in this respect?
- The next questions look at your own approach in treating dementia: Which **intervention approaches** do you consider sensible and effective at slowing down the progression of dementia, and do you practise them yourself? Please give your reasons.
- What **positive effects** have you already seen in this respect? This refers to influence on cognitive abilities, quality of life for patients and relatives and so on.
- In your experience, **how easy or difficult** is it for a general practitioners to provide **treatment and management** for dementia patients? Please give reasons.
- What **challenges or problems** do you see in managing dementia patients? Could you describe your experiences and observations here?
- What role do **relatives** play in the treatment management process? How can we involve them in the process?
- From your experience**, how well do you think general practitioners and specialists** (neurologists; general and gerontological psychiatrists) **work together** in therapeutic management of dementia patients? What do you think works well, what doesn’t so much?
- How often do you as a general practitioner have a completely **different point of view** or approach from a specialist consultant in treating dementia? Why do you think this might be so?
- What have your experiences been in **interdisciplinary work** on **drug treatment** for dementia?
- In your opinion: How familiar are you with **regional supply structures** providing counselling and support for dementia patients and their relatives? Examples include services from care centres and dementia networks.
- To what extent do you work with these or other **institutions and services** in treating dementia patients?
- How effective is collaboration and **timely guidance** for patients and relatives in **stabilising** the disease?
- Do you think more intense **collaboration with other institutions and services would be beneficial**? Which institutions?
- Does **dementia prevention**, that is, treating dementia after diagnosis, play **the role** it should in treatment? Please state any reasons why or why not.
- What **approaches** do you think would be beneficial towards **improving and promoting** awareness specifically for dementia prevention? What support would specifically benefit general practitioners in taking charge of therapeutic management and making it more effective? Examples might include improvement in early detection, structured care programmes with regular patient presentations, targeted further training and training formats.
- How would you rate your own **knowledge and capabilities** in treating dementia? How well versed are you in the subject? Where do you feel sure and where do you feel less sure? What else would you like to learn?
- Have you already **attended an advanced training course** (or more) with dementia as a relevant topic? What training courses were they? How did they benefit you – what appealed to you, what didn’t appeal to you so much?
